# Assessing the effect of temperature on Rhodococcus metabolite production

**DOI:** 10.1099/mic.0.001598

**Published:** 2025-08-26

**Authors:** Marla I. Macias-Contreras, Natalie Millán-Aguiñaga, Jonathan Parra, Katherine R. Duncan

**Affiliations:** 1 Strathclyde Institute of Pharmacy and Biomedical Sciences, University of Strathclyde, Glasgow, G4 0RE, UK; 2 Facultad de Ciencias Marinas, Universidad Autónoma de Baja California, Ensenada, Baja California, México; 3 Centro de Investigaciones en Productos Naturales (CIPRONA), Universidad de Costa Rica, San José 11501-2060, Costa Rica; 4 Centro Nacional de Innovaciones Biotecnológicas (CENIBiot), CeNAT-CONARE, San José 1174-1200, Costa Rica; 5 Biosciences Institute, Newcastle University, Newcastle upon Tyne, NE2 4HH, UK

**Keywords:** actinomycetes, environmental microbiology, *Rhodococcus*, specialized metabolites

## Abstract

Rare actinomycetes are increasingly recognised as a valuable yet underexplored source of bioactive compounds with significant biomedical potential. While it is well established that bacteria have evolved adaptive mechanisms to withstand environmental stressors, such as variations in temperature, salinity or pH, our understanding of how these abiotic parameters influence bacterial metabolism remains limited. This has important implications not only for laboratory cultivation but also for predicting microbial behaviour in natural ecosystems. In this study, we investigated the effect of temperature on specialized metabolite production by the genus *Rhodococcus*. Seven strains isolated from marine sediments in three regions – Scotland, the sub-Arctic and Antarctica – were cultured at 20, 25 and 30 °C. Strain-specific growth curves were generated to normalize metabolite extraction at equivalent growth stages, resulting in a total of 54 *Rhodococcus* metabolite extracts. Liquid chromatography-high-resolution mass spectrometry analysis combined with molecular networking revealed that lower cultivation temperatures reduced bacterial biomass and delayed the onset of the stationary phase, and strain *Rhodococcus* KRD197 exhibited temperature shifts in metabolism that were associated with alterations in carbohydrate and fatty acid metabolism, potentially linked to osmotic regulation and cell membrane adaptation. These findings highlight the impact of temperature on *Rhodococcus*-specialized metabolism and support the potential of rare actinomycetes from extreme environments for expanding chemistry from these understudied genera.

## Data Availability

Raw MS-MS data have been deposited in MassIVE under accession number MSV000098160. All supporting data for this study, including growth curves, molecular networks and analysis underlying Figs 2, 3, 4 and S1–S9, have been deposited in Figshare. All datasets are grouped under the project titled *Assessing the effect of temperature on Rhodococcus metabolite production*. GNPS Feature-Based Molecular Networking results for *Rhodococcus* strains under variable temperature conditions: https://figshare.com/s/1eb9369d4aaea7a697fb. Annotation data by SIRIUS of all *Rhodococcus* strains at 20, 25 and 30 °C: https://figshare.com/s/8226456c7b7b5d9db265. GNPS and DEREPLICATOR annotations and networks for KRD197 strain: https://figshare.com/s/28ea6f9e2996d173af75. Annotation data by SIRIUS of strain KRD197 at 20, 25 and 30 °C: https://figshare.com/s/752053eea7c9b16cee02. OD over time of six *Rhodococcus* strains (KRD162, 197, 226, 231, 175 and *Rhodococcus fascians*): https://figshare.com/s/c9da4531eb2a7c4956c2.

## Introduction

Antibiotic resistance represents one of the most serious threats to global health, responsible for an estimated 1.27 million deaths worldwide in 2019, with an additional 4.95 million deaths associated with drug-resistant infections worldwide [1,2]. This growing crisis underscores the urgent need for novel bioactive compounds to combat multidrug-resistant pathogens [3]. Historically, members of the order *Actinomycetales* (commonly referred to as actinomycetes, an order within the phylum *Actinomycetota*) have been the predominant source of antibiotics, with the genus *Streptomyces* alone estimated to contribute to ~55% of all antibiotics discovered between 1945 and 1978 [4,5]. However, the extensive exploration of this group has led to a decline in the discovery of new molecules [6]. In response, attention has shifted towards rare actinomycetes, which represent a promising and underexplored reservoir of specialized metabolites with significant therapeutic potential. One notable example is fidaxomicin, an FDA-approved antimicrobial used in the treatment of *Clostridium difficile* infections, which was isolated from *Dactylosporangium aurantiacum* subsp. *hamdenesis* [7].

The biosynthesis of specialized metabolites is achieved by enzymes that are encoded by biosynthetic gene clusters (BGCs), which contain the necessary genes for synthesis, regulation and transport of these metabolites [8]. Advances in sequencing have revealed that many members of the phylum *Actinomycetota* harbour substantial biosynthetic potential that remains largely untapped [9,10]. A recent (meta)genomic study identified *Streptomyces* as the most biosynthetically diverse genus within the phylum, while also highlighting other genera, such as *Amycolatopsis*, *Mycobacterium*, *Nocardia* and *Micromonospora*, as underexplored sources of chemical novelty [11]. Among these, the genus *Rhodococcus* stands out for its metabolic versatility, industrial relevance and growing recognition as a producer of specialized metabolites, including antibiotics [12]. For instance, *Rhodococcus jostii* K01-B0171 produces lariatin A and B, cyclic peptides with strong antibacterial activity against *Mycobacterium smegmatis* and *Mycobacterium tuberculosis* [13,14], and *Rhodococcus opacus* R7 produces a novel biosurfactant with antimicrobial activity against *Escherichia coli* and *Staphylococcus aureus* [15]. Despite these findings, the biosynthetic potential of *Rhodococcus* remains largely unexplored. As of March 2025, 934 *Rhodococcus* genomes have been deposited in the National Center for Biotechnology Information (NCBI), with 143 categorized as complete or chromosomal level [16]. According to the antiSMASH database [17], a total of 4,503 BGCs are associated with this genus: 43% correspond to non-ribosomal peptide synthetases, 16% to ribosomally synthesized and post-translationally modified peptides, 14% to polyketide synthases and 12% to terpenes, among others. This highlights the vast, yet largely uncharacterized, biosynthetic potential of *Rhodococcus*, as demonstrated in recent genome-wide BGC analyses [9,10].

However, a major limitation in natural product discovery is the ‘silent’ nature of many BGCs under standard laboratory conditions, suggesting that it may be environmental triggers that are necessary for BGC expression. In fact, it is estimated that only 3% of predicted BGCs are correlated with experimentally verified metabolites [11]. One promising strategy to overcome the challenge of silent BGCs is the exploration of micro-organisms from extreme environments, which may be adapted to their extreme surroundings. For example, Millán-Aguiñaga *et al*. identified rare actinomycetes from Polar sediments, including species from the genera *Microbacterium*, *Rhodococcus* and *Pseudonocardia* [18]. Further studies by Parra *et al*. confirmed the presence of novel *Pseudonocardia* species from these environments [19]. These Polar ecosystems have garnered increased attention due to their extreme ecological conditions – low temperatures, high UV radiation and limited nutrients – which may drive the evolution of specialized metabolic pathways. For example, *Deschampsia antarctica*, an endemic Antarctic plant, has been identified as a reservoir of bioactive *Actinomycetota* including diverse genera such as *Actinoplanes*, *Arthrobacter*, *Kribbella*, *Mycobacterium*, *Nocardia*, *Pilimelia*, *Pseudarthrobacter*, *Rhodococcus*, *Streptacidiphilus*, *Streptomyces* and *Tsukamurella*, with some showing antitumour activity [20]. In the laboratory, environmental parameters such as salinity, nutrient availability (including trace metals) and temperature have been manipulated to elicit the expression of silent BGCs. For example, *Streptomyces lunaelactis* regulates the production of ferroverdins and bagremycins in response to iron availability where the antimicrobial bagremycins are biosynthesized under iron-limited conditions, while excess iron promotes ferroverdin production, a siderophore with pharmaceutical applications [21]. To facilitate metabolite comparison across conditions and strains, several comparative metabolomic approaches have been used such as molecular networking (GNPS) [22], SIRIUS [23] and MetaboAnalyst [24]. For example, Riccardi *et al*. demonstrated that changes in cultivation temperature modulated the metabolite profiles of a cold-adapted marine bacterium, indicating that temperature can indeed elicit the expression of certain biosynthetic pathways [25].

Understanding how abiotic factors, such as temperature, influence the metabolic responses of rare actinomycetes from underexplored ecosystems is likely crucial for unlocking their biosynthetic potential. In this study, we investigated the effect of temperature on the specialized metabolite production of *Rhodococcus* strains isolated from Scottish and Polar marine sediments.

## Methods

### Sediment collection

Sediment samples were collected from three geographically distinct regions: Scotland, Antarctica and the Arctic (Table S1, available in the online Supplementary Material). Scottish samples were collected aseptically by hand from the shoreline of Oban Bay using a sterile 50-ml centrifuge tube. Samples were subsequently preserved on ice for transport and frozen at −20 °C until further processing. Sediment samples from the Antarctic and Arctic regions were obtained during research expeditions aboard the PS Polarstern (2002) and RRS James Clark Ross (2005) cruises [18].

### Bacterial isolation

Polar sediments were processed following the protocol detailed by Millán-Aguiñaga *et al*. [18]. Scottish sediments were pre-treated, and bacterial isolation was performed using the dry/stamp (DS) method as described by Jensen *et al*. (DS) [26]. Briefly, 1 g of sediment was placed in a sterile Petri dish and dried in a laminar flow hood overnight. Subsequently, the sediment was stamped with a sterile sponge (20 mm diameter) and inoculated on starch casein (SC) nitrate agar (10 g soluble starch, 1 g sodium caseinate, 2 g KNO_3_, 0.5 g KH_2_PO_4_, 0.5 g MgSO_4_, 18 g Instant Ocean and 18 g agar) at pH 7.8–8.30 [27]. To inhibit the growth of fungi and Gram-negative bacteria, the isolation media were supplemented with 25 µg ml^−1^ cycloheximide, 25 µg ml^−1^ nystatin and 10 µg ml^−1^ nalidixic acid. The culture plates were incubated at room temperature for 3–4 weeks. Colonies displaying morphological characteristics of actinomycetes were sub-cultured onto fresh SC agar until pure colonies were obtained. All strains were cryopreserved in 25% glycerol (v/v) and stored at −80 °C.

All *Rhodococcus* strains from the Duncan lab bacterial culture collection selected for analysis (Table S1) were inoculated onto ISP2 agar [28], supplemented with 18 g l^−1^ Instant Ocean® Sea Salt (IO) (Instant Ocean, USA) and incubated for 7 days at 30 °C before routine culture.

### Antibacterial assays using an agar plug diffusion method

Antimicrobial assays were performed following a previously published method [29,30]. A panel of ESKAPE pathogens (*Enterococcus faecalis*, *Staphylococcus aureus*, *Klebsiella pneumoniae*, *Acinetobacter baumannii* and *Pseudomonas aeruginosa*) and additionally *E. coli* and *Bacillus subtilis* was cultured using Lysogeny Broth medium (peptone 10 g l^−1^, yeast extract 5 g l^−1^ and sodium chloride 5 g l^−1^) [5 ml, 30 °C, 82.1×g (1,200 r.p.m.), 12 h]. These were used to inoculate nutrient agar (NA, 25 ml per plate), with the pathogen adjusted to an OD of 0.01 before plating. Previously, all *Rhodococcus* strains were grown on ISP2 agar for 7 days [30]. An agar disc (7 mm) of the actinomycete lawn was then transferred to the NA pathogen culture and incubated (overnight, 30 °C) before measuring the zone of inhibition around the actinomycete plug (mm) [31]. An ISP2 agar disc was used as a negative control, and a sterile paper disc with 10 µg µl^−1^ gentamicin was used as a positive control.

### DNA isolation, amplification and sequencing of the 16S rRNA gene

All *Rhodococcus* strains were cultivated in ISP2 broth [28] at 30 °C with constant agitation (220 r.p.m.) for 7 days. Bacterial cells were concentrated by centrifugation [9,633 ***g*** (13,000 r.p.m.)×15 min], and the genomic DNA was extracted using the salting out method from Kieser *et al*. [32] and Feeney *et al*. [33]. DNA was quantified by NanoDrop, visualized to determine its quality (high molecular weight) by agarose gel (1%) electrophoresis and stored at −20 °C. 16S rRNA gene amplification by PCR was achieved using universal primers FC27 (5′-AGAGTTTGGATCMTGGCTCAG-3′) and RC1492 (5′-CGGTTACCTTGTTACGACTT-3′) [34] to generate a PCR product of 1,500 bp. PCR conditions were as follows: denaturation at 95 °C for 15 min followed by 32 cycles at 95 °C for 1 min, 61 °C for 1 min and 72 °C for 1 min, ending with a final extension at 72 °C for 7 min [35]. Amplicons were verified by agarose gel electrophoresis (1% agarose) with a 1,000 bp reference ladder (BioLabs), using distilled water as a negative control, while pure genomic DNA from a *Rhodococcus* strain served as a positive control. Amplicons were purified using a QIAquick PCR cleanup kit (QIAGEN Inc., Manchester, UK) according to the manufacturer’s protocol and sent to Eurofins Genomics (Germany) for sequencing. The obtained 16S rRNA gene sequences were manually inspected and trimmed with BioEdit sequence alignment editor [36], and multiple alignment was achieved using the blast tool in the NCBI platform [37] (Table S2).

### Phylogenetic analysis

Phylogeny inference was performed using the Genome to Genome Distance Calculator (GGDC) web server [38] using the DSMZ phylogenomic pipeline [39] adapted to single genes. A multiple sequence alignment was created with muscle [40]. Maximum likelihood (ML) and maximum parsimony (MP) trees were inferred from the alignment using RAxML [41] and TNT [42], respectively, using the GTR+GAMMA substitution model. For ML, rapid bootstrapping in conjunction with the autoMRE bootstrapping criterion [43] and subsequent search for the best tree was used; for MP, 1,000 bootstrapping replicates were used in conjunction with tree-bisection-and-reconnection branch swapping and 10 random sequence addition replicates. The sequences were checked for a compositional bias using the Χ² test as implemented in PAUP* [44]. For phylogenetic tree visualization and post-modification, FigTree v1.4.3 was used [45].

### *Rhodococcus* culture at 20, 25 and 30 °C

*Rhodococcus* strains were pre-cultured (ISP2 broth [30], 10 ml, 30 °C, 250 r.p.m., 7 days) and used to inoculate (2.5 ml) cultures in triplicate (50 ml, ISP2 broth). ISP2 medium was chosen as it supported the cultivation of all strains in this study, enabling direct comparison of metabolite production across temperatures. For each strain, the inoculated flasks were incubated at three temperatures (20, 25 and 30 °C), with a total of nine flasks per strain, resulting in 54 flasks for all six strains. A media blank (no bacterial culture added) was included in triplicate at each temperature (nine flasks total). Four hours after inoculation, 1 ml was taken from each flask and transferred to a disposable spectrophotometer cuvette to measure the absorbance at 600 nm (Jenway 6300, UV/Vis Spectrophotometers). Aliquots were collected every 12 h after inoculation for 168 h (7 days) [46].

### *Rhodococcus* growth measurement

Growth curves were generated by plotting the average OD (with OD values that were first multiplied by 100 to account for sample dilution) of each triplicate sample against time (hours), with sd represented as vertical error bars [46]. The onset of the stationary phase was determined using GraphPad Prism 8.0.1 [47]. The specific growth rate (*μ*) was calculated using equation 1, applied to the log-transformed OD values corresponding to the exponential growth phase, as shown in Fig. S1. Additionally, a t-test with false discovery rate correction (Benjamini, Krieger and Yekutieli, *Q*=1%) was performed in GraphPad Prism 8.0.1 [47] to assess statistically significant differences of growth rates between temperatures.


(1) $$\mu =[\text{ln}(N_{t}))-\text{ln}(N_{0})]/(t-t_{0})*100$$

*µ* is the specific growth rate (%).

*N*_0_ is the biomass at the beginning of the exponential growth phase (g).

*N*_t_ is the biomass at the end of the exponential growth phase (g).

*t*−*t*_0_ is the duration of the exponential phase (days).

In this study, OD_600_ values were used as a proxy for biomass (*N*).

### Metabolite extraction of bacterial cultures

Metabolite extraction was performed 24 h after each culture reached stationary phase. To facilitate metabolite adsorption, HP-20 resin (2.5 g) was added to each flask 2 h prior to extraction, following the protocols routinely used in our lab [18,48]. After 2 h, this was centrifuged [15 min, 4 °C and 4,140 ***g*** (5,000 r.p.m.)], the supernatant was discarded and the cell/resin pellet was frozen at −80 °C overnight. The cell/resin pellet was then lyophilized (until completely dry, approx. overnight), transferred to an Erlenmeyer flask and extracted twice with ethyl acetate (20 ml, VWR HPLC grade) for 2 h at 250 r.p.m.; as such, the extraction was biassed towards moderately polar to lipophilic compounds. The extracts were then combined and evaporated under nitrogen, weighed and stored at 4 °C [48]. Solvent and medium samples (no bacteria) were included as controls.

### Liquid-chromatography-high resolution tandem mass spectometry analysis of crude extracts

The extracts were resuspended in acetonitrile (HPLC grade, MERCK, UK) to a concentration of 1 mg ml^−1^ and analysed by liquid chromatography-high-resolution MS (LC-HRMS/MS) using a Dionex UltiMate™ 3000 coupled to Q-Exactive™ (Thermo Scientific, Germany) mass spectrometer with an electrospray ionization source and a mass range of 100–1050 *m/z* in positive ionization mode with a spray voltage of 1.5 kV and capillary temperature at 250 °C. An Accucore™ C18 (100×2.1 mm) column was used for chromatographic separation at 45 °C. Mobile phase A consisted of H_2_O with 0.1% formic acid (Fluka® Analytical, Switzerland), and mobile phase B consisted of acetonitrile Optima™ LC/MS (Fisher Scientific, UK) with 0.1% formic acid. A gradient was used starting from 1% B and increasing to 50% at 2 min and then to 99% at 10.5 min, and it was held constant for 0.5 min. Finally, B was brought back to initial conditions and held for 5 min for a total run time of 15 min. A flow rate of 300 µl min^−1^ and an injection volume of 5 µl were used. MS-MS was performed in data-dependent acquisition mode at a resolution of 17,500 (at *m/z* 200) (standard settings for Q Exactive Orbitrap), using an isolation window of 1 *m/z* with no offset. Stepped collision energies were applied at 20, 30 and 40 eV.

### Data pre-processing and peak detection

The raw MS data were first converted to mzML file format using Proteowizard MSConvert [49], ensuring compatibility for processing in MZmine3 [50]. Using MZmine, the following specifications were followed: mass peaks were detected in positive mode using a centroid mass detector to identify and extract the signal peaks from raw data based on their mass-to-charge ratio (*m/z*). The noise level was set to 1.0×10^6^ for MS1 data (precursor ions) and 1.0×10^3^ for MS2 data (fragment ions) to exclude low-intensity signals likely to represent background noise. Chromatograms were constructed using the ADAP module [51], which links detected peaks over time to form chromatographic features. Parameters included a minimum maximum intensity of 5.0×10^4^ and an *m/z* tolerance of 0.002. To separate overlapping peaks into distinct features, the chromatograms were deconvoluted using the minimum local baseline search algorithm with a minimum retention time range of 0.05 min, a minimum relative height of 0% and a minimum absolute height of 5.0×10^4^. Finally, a peak alignment list was created using the Join Aligner module, with *m/z* tolerance of 0.0015, a retention time tolerance of 0.1 min and weights for a retention time of 1 and for a *m/z* of 3.

### Statistical analysis and visualization of filtered peaks

The final list of molecular features was exported as a .csv file for statistical analysis and as fragmentation patterns (MS2), while spectral data were exported as a .mgf file for metabolite annotation and classification into chemical classes, enabling compatibility with bioinformatic tools and facilitating both quantitative and qualitative analyses. The statistical analysis included both univariate and multivariate approaches. The cyclic Loess algorithm was implemented in NormalyzerDE (http://quantitativeproteomics.org/normalyzerde) to ensure that the replicate groups were normalized independently, reducing technical variation and enabling meaningful comparisons across experimental groups [52]. Univariate statistical analysis was achieved through volcano plots using MetaboAnalyst 6.0 [53] to identify features with statistically significant differences between groups. This approach combined fold change analysis and statistical significance (e.g. *P*-value) to highlight key metabolites of interest. Multivariate statistical analysis and visualization were achieved using hierarchical grouping and heatmap visualization with MetaboAnalyst 6.0. Before creating heatmaps, the data were filtered to exclude features with a relative sd greater than 25%, ensuring reliability. The data were then transformed to a Log 2 scale, which stabilizes variance and enhances the interpretability of patterns in the dataset.

### Molecular networking

A molecular network was created with the Feature-Based Molecular Networking (FBMN) workflow [22] on GNPS (http://gnps.ucsd.edu) [54] using .mzML files. The MS data were first processed with MZmine3 [50], and the results were exported to GNPS for FBMN analysis. The data were filtered by removing all MS-MS fragment ions within ±17 Da of the precursor *m/z*. MS-MS spectra were window-filtered by choosing only the top six fragment ions in the ±50 Da window throughout the spectrum. The minimum fragment ion intensity in the MS-MS spectra was set to 1,000. The precursor ion mass tolerance was set to 0.02 Da and the MS-MS fragment ion tolerance to 0.02 Da. A molecular network was then created where edges were filtered to have a cosine score above 0.7 and more than six matched peaks. Further, edges between two nodes were kept in the network if and only if each of the nodes appeared in each other’s respective top ten most similar nodes. Finally, the maximum size of a molecular family was set to 100, and the lowest-scoring edges were removed from molecular families until the molecular family size was below this threshold. The spectra in the network were then searched against GNPS spectral libraries [54,55]. The library spectra were filtered in the same manner as the input data. All matches kept between network spectra and library spectra were required to have a score above 0.7 and at least six matched peaks. The DEREPLICATOR was used to annotate MS-MS spectra [56]. The molecular networks were visualized using Cytoscape software [57].

### Metabolite annotation

The *in silico* structure annotation was performed using SIRIUS software (v5.8.6) [23], integrating CSI:FingerID [58] for structure prediction and CANOPUS [59] for chemical class assignment based on NPClassifier ontology [60]. Only compounds with a molecular weight <850 Da were included to reduce computational time.

Annotations were based on fragmentation spectra, isotope patterns and exact mass. CSI:FingerID predictions were retained if the structure match score exceeded 90%, and CANOPUS chemical class assignments were considered reliable when class probability scores exceeded 0.7. No custom settings were applied beyond the default mass accuracy parameters provided by SIRIUS. In both FBMN and SIRIUS workflows, biological replicates were treated as independent samples.

## Results

### *Rhodococcus* phylogeny

**Fig. 1.**
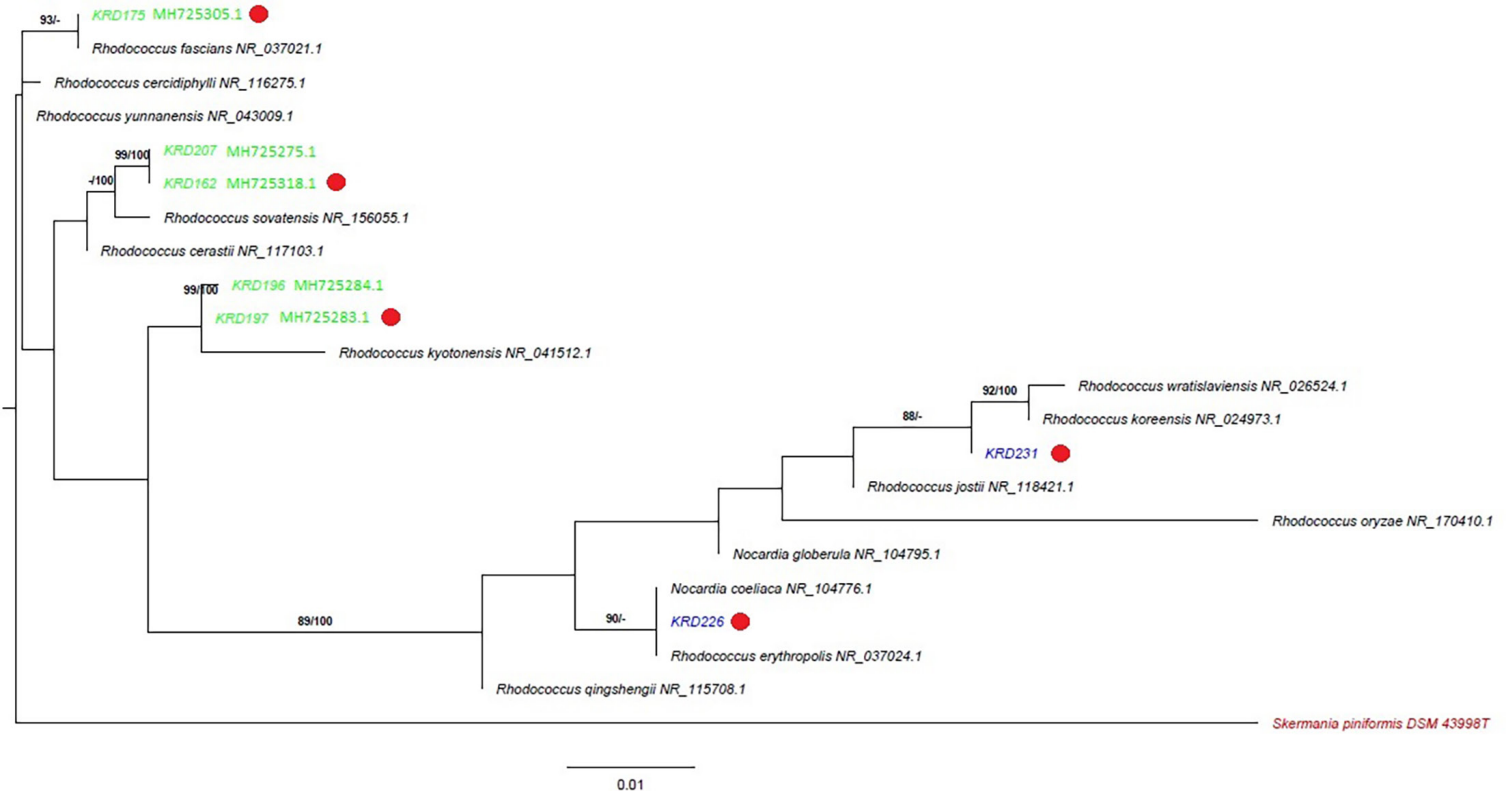
ML and MP tree based on 16S rRNA gene sequences of seven *Rhodococcus* strains isolated from the Arctic and Antarctic. This shows the genetic distance between isolates and closely related type strains (<96% similarity, NCBI accession numbers) with *Skermania piniformis* DSM 43998 used as an outgroup. The branches are scaled in terms of the expected number of substitutions per site; the scale bar represents 0.01 substitutions per site. The numbers above the branches are support values exceeding 60% of the ML (left) and MP (right) bootstrap. Strain origin is represented by colours: green, Arctic/Antarctic; blue, Scotland; and red, outgroup. The red dot indicates that these strains were selected for further analysis.

The 16S rRNA gene-based phylogeny of the seven selected strains (Fig. 1), comprising five isolates from Polar sediment and two from Scottish sediment (Table S1), revealed clustering into two well-supported clades, where the strains from the Arctic–Antarctic area clade with the type strains *Rhodococcus fascians* NR_037021.1 and *Rhodococcus yunnanensis* NR_043009.1 (bootstrap 99–100%), while the strains from Scotland form a second clade with type species such as *Rhodococcus erythropolis* NR_037024.1 and *Nocardia coeliaca* NR_104776.1 (reclassification of the latter to *Rhodococcus* has been suggested [61,62]) (bootstrap ≥88%). This geographic separation into two clades is noteworthy; however, it is important to emphasize that 16S rRNA gene sequences alone lack sufficient resolution to reliably resolve phylogenetic relationships within closely related actinomycetes. Additionally, the limited sample size restricts the ability to draw firm conclusions regarding species divergence based on geographic origin. Based on phylogenetic diversity and morphological characteristics, five strains of *Rhodococcus* were selected for further metabolomic and phenotypic analyses (KRD162, KRD197, KRD175, KRD226 and KRD231). As part of the strain selection process, antimicrobial activity against the ESKAPE pathogens was used as an initial screening criterion to identify promising bioactive producers. However, none of the strains exhibited detectable antimicrobial activity. This preliminary screening was conducted using agar plug assays from strains grown on ISP2 agar at 28 °C for 7 days (data not shown).

### Growth dynamics and determining stationary phase

**Fig. 2.**
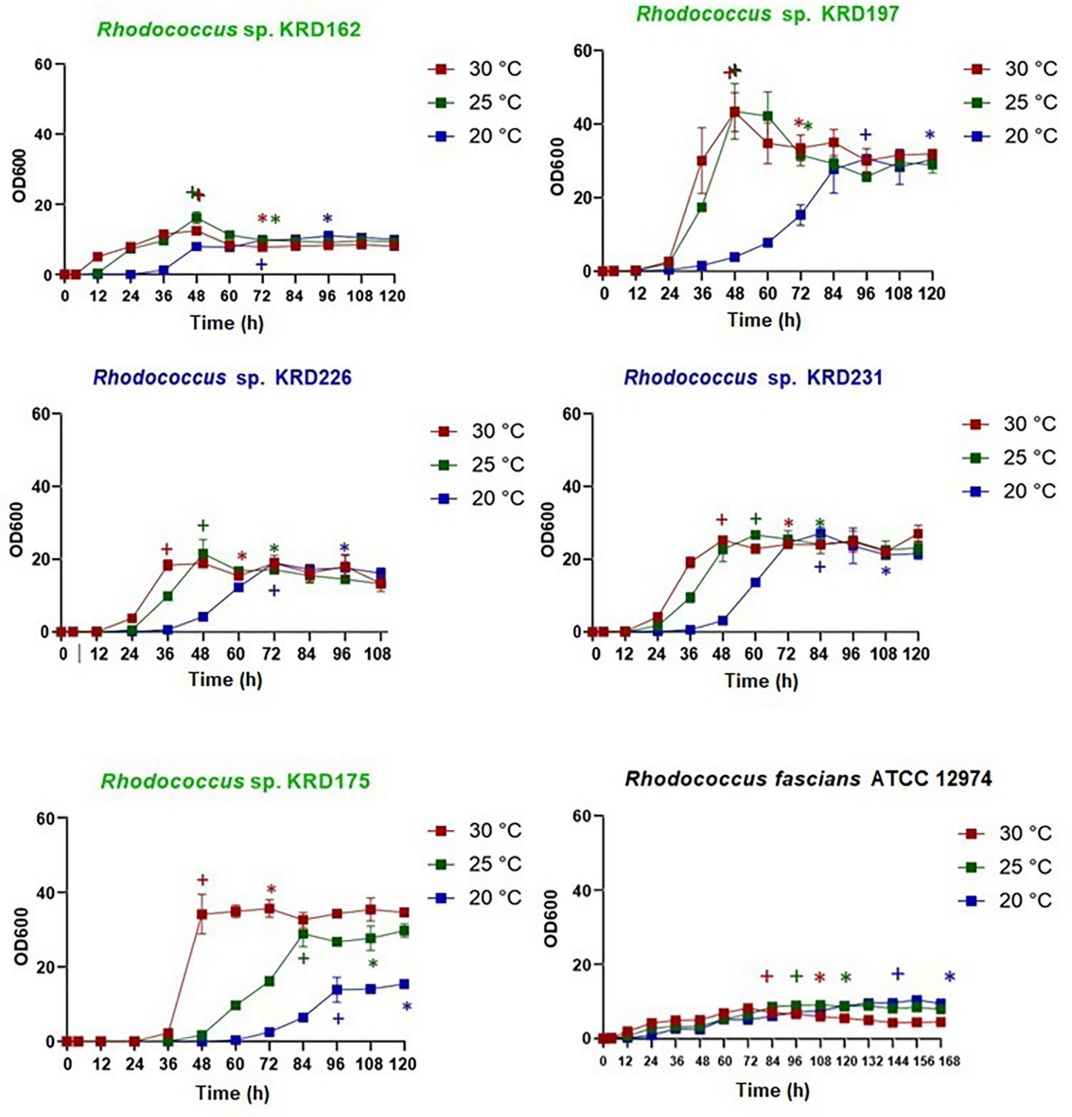
OD over time of six *Rhodococcus* strains (KRD162, 197, 226, 231, 175 and *R. fascians* ATCC 12974) cultured at 20, 25 and 35 °C in ISP2 medium. The time (hours) is on the x-axis and OD at 600 nm on the y-axis. Data points represent the average value of three replicates. Error bars represent the sd of triplicate cultures. The origin of the strains is represented by the colour of the graph title: green, Arctic/Antarctic; blue, Scotland; and black, *Rhodococcus* type strain. A cross indicates the start of the stationary phase and an asterisk the selected extraction point.

To assess the impact of temperature on metabolite production, standardization of the extraction point was required to ensure that the growth stage was consistent across strains. For this purpose, five *Rhodococcus* strains (KRD162, KRD197, KRD175, KRD226 and KRD231) and one type strain (*R. fascians* ATCC 12974) were cultured in triplicate at three temperatures: 30, 25 and 20 °C. Although additional experiments were initially conducted at lower temperatures (10 and 15 °C), the growth of all strains – including those from Polar environments – was too slow and insufficient to generate reproducible growth curves or extract metabolites for analysis. These conditions were therefore excluded from the study. All strains showed clear growth phases at all temperatures (lag, exponential growth and stationary phases) (Fig. 2). In general, the lag phase lasted between 0 and 36 h, the exponential phase occurred between 4 and 132 h and the stationary phase was reached between 48 and 132 h, depending on the strain and temperature. For example, *Rhodococcus* KRD162 and KRD197 reached the stationary phase at 48 h at 30 and 25 °C, while these same strains reached the stationary phase at 20 °C at 72 and 84 h, respectively. The results showed that although strains KRD162, KRD197 and KRD175 were isolated from a Polar environment, there was no relationship between temperature and origin. For example, strains KRD197 and KRD175 reached the highest cell densities, of around OD_600_=0.4 x 100 = 40, while KRD162 consistently remained below 20. The calculation of the specific growth rate (%*−μ*/day) for each strain (Fig. S2), based on the natural logarithm of the linear portion of the growth curves, revealed that strains KRD197, KRD175, KRD226 and KRD231 followed the expected trend: their growth rate increased at higher temperatures, meaning that cell division occurred at a faster rate, leading to a shorter lag phase and an earlier transition to the stationary phase. In contrast, at lower temperatures, the growth rate decreased, resulting in a prolonged lag phase and a delayed entry into the stationary phase. However, strain KRD162 showed atypical behaviour, growing slower at 30 °C and reaching a higher cell density at 25 °C. Statistical analysis (t-test) showed that the specific growth rates of strains KRD162, KRD175 and KRD226 were significantly different across all three temperatures tested. KRD197 and KRD231 displayed significantly lower growth rates at 20 °C compared with 25 and 30 °C (Fig. S2). In contrast, strain *R. fascians* ATCC 12974 did not exhibit significant differences in growth rate across the temperatures tested. These results suggest that although these strains were isolated from cold or temperate marine regions, their temperature responses are strain-specific and do not necessarily reflect a uniform adaptation to low temperatures. With the growth established for each strain at three temperatures, the metabolite extraction point was set at 24 h after reaching the stationary phase, ensuring that all strains would be in the same growth phase for comparison of their specialized metabolites. According to several reports, the production of antibiotics by actinomycetes occurs mainly after the stationary phase is reached, and as such, this was chosen to maximize chemical diversity.

### Molecular network analysis of *Rhodococcus* strains highlights the distinct metabolic profile of KRD197

**Fig. 3.**
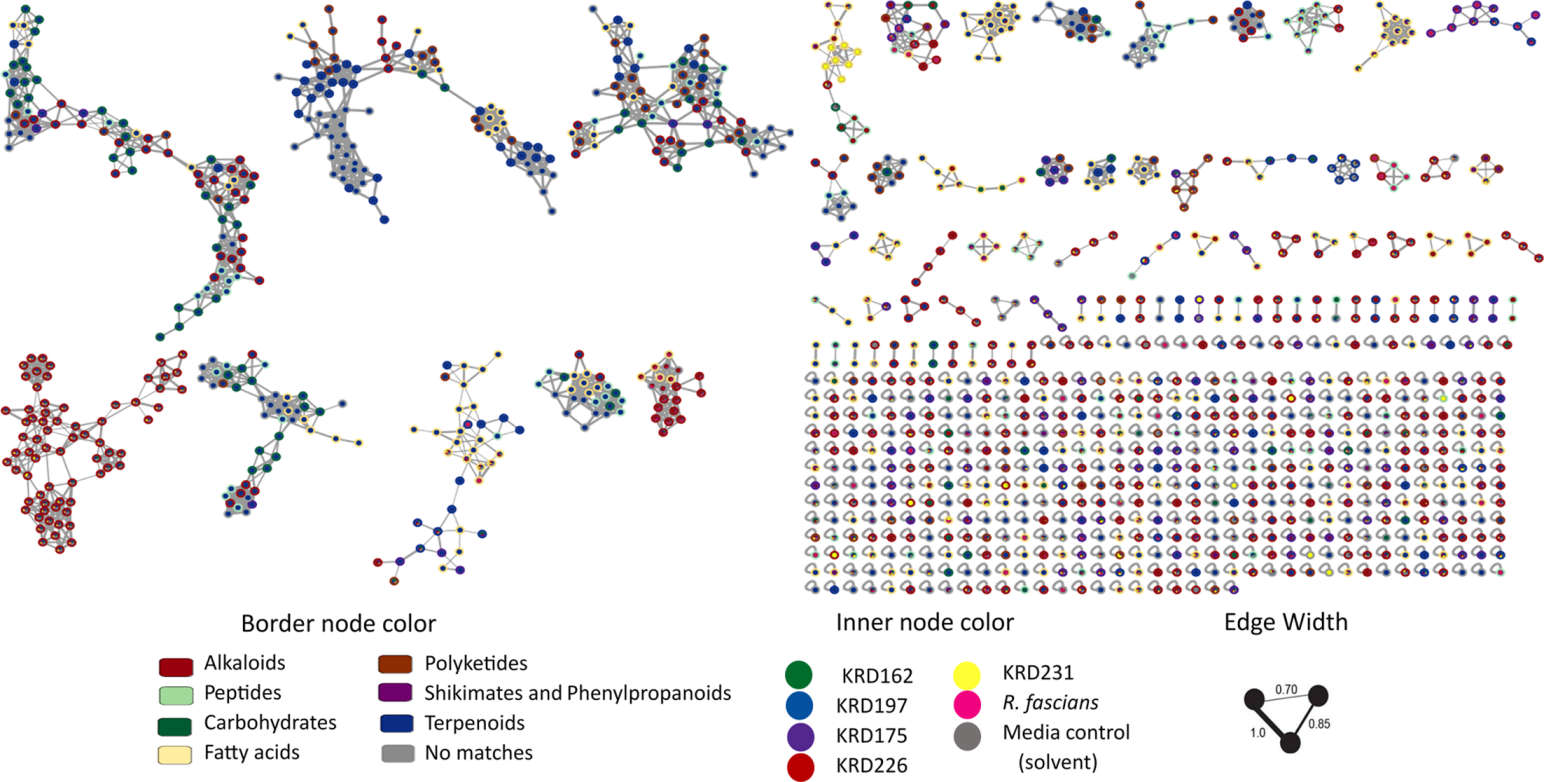
FBMN of six *Rhodococcus* strains at 20, 25 and 30 °C. Inner pie chart node colour represents the presence of each feature across the strains, and grey represents features present in the culture solvent/media controls. Border node colour represents the chemical class annotation, and parent ions without matches are coloured grey.

Metabolite extracts were obtained from the cultures of all six strains after 24 h in the stationary phase and analysed by LC-HRMS/MS in positive ionization mode. The resulting MS-MS data were uploaded to the GNPS platform for molecular networking analysis. The molecular network constructed from this data consisted of 1,280 features (i.e. distinct molecular ions) (Fig. 3), organized into 86 molecular families (groups of 2 or more nodes connected by edges due to similar fragmentation patterns). These features were classified into seven biosynthetic categories, alkaloids, aa and peptides, carbohydrates, fatty acids, polyketides, shikimates and phenylpropanoids and terpenoids, using CANOPUS [59] and the NPClassifier ontology [60]. It should be noted that the metabolite profiles obtained in this study reflect only the compounds extractable with ethyl acetate under the conditions used. As such, the analysis is inherently biassed towards moderately polar to lipophilic metabolites and may exclude highly polar or volatile compounds. Additionally, while the HP-20 resin has shown high affinity for a broad range of bacterial metabolites in previous studies [63,64], its capture efficiency was not directly evaluated here. These methodological choices were made to prioritize the detection of drug-like small molecules and ensure consistency with our laboratory’s established workflows. Among these, the fatty acid pathways represented the largest proportion of features, accounting for 18.5% of the total. Additionally, 467 features (36.5%) were identified as singletons (fragmentation patterns not correlated with others), suggesting the presence of chemical diversity within the samples. In contrast, 434 features (33.9%) were present in both media and solvent controls (grey nodes), indicating that they originated from media components.

Among the analysed *Rhodococcus* strains, KRD197, KRD226 and KRD175 had the highest number of features, with 1,095 (85.5%), 565 (44.14%) and 513 (40.07 %) features, respectively. Notably, KRD197 exhibited 632 exclusive features, underscoring its distinct metabolic profile (Fig. S3). However, the distribution of features across all strains did not follow a defined pattern in relation to temperature, suggesting that metabolite production by *Rhodococcus* is primarily species-specific, rather than phylogenetic or geographical. For example, strains from Arctic–Antarctic environments shared 364 features with their most closely related type strain, *R. fascians*, while strains isolated from Scotland shared 385 features with this species, despite being more distantly related.

### Temperature-dependent metabolite production by *Rhodococcus* KRD197

*Rhodococcus* KRD197 exhibited the highest number of features (1,095) among the analysed strains (Fig. S7A) and was therefore selected to evaluate the effect of temperature on metabolite production. The molecular network generated from this particular strain had 1,095 features, distributed across 57 molecular families (68.6%) and 344 singletons (31.4%), highlighting its chemical diversity. The majority of these features (819) were observed at 20 °C, followed by 416 at 30 °C and 374 at 25 °C (Fig. S7B). The higher number of features at lower temperatures suggests that the strain may activate defensive metabolic pathways in response to stress induced by colder environments.

In general, the proportions of natural product categories varied with temperature. Carbohydrates and polyketides showed a marked increase as the temperature decreased, with their proportions rising from 1% and 7%, respectively, at 30 °C, to 11% and 12%, respectively, at 20 °C. Peptides also increased from 6% at 30 °C to 10% at 20 °C (Fig. S6). This activation of the carbohydrate biosynthetic pathway has been associated with enhanced stress resistance in other species.

Interestingly, KRD197 was the only strain that showed an increase in the number of features as the temperature decreased (Fig. S4). This strain also exhibited a higher proportion of features across all chemical classes compared with other strains, which maintained a similar number of features across chemical classes regardless of their geographical origin (Scottish or Polar, Fig. S5). These findings suggest that KRD197 may possess adaptive mechanisms that respond to temperature-induced stress, potentially activating biosynthetic pathways that lead to the production of a broader range of metabolites.

### *Rhodococcus* KRD197 showed metabolic shifts induced by temperature

**Fig. 4.**
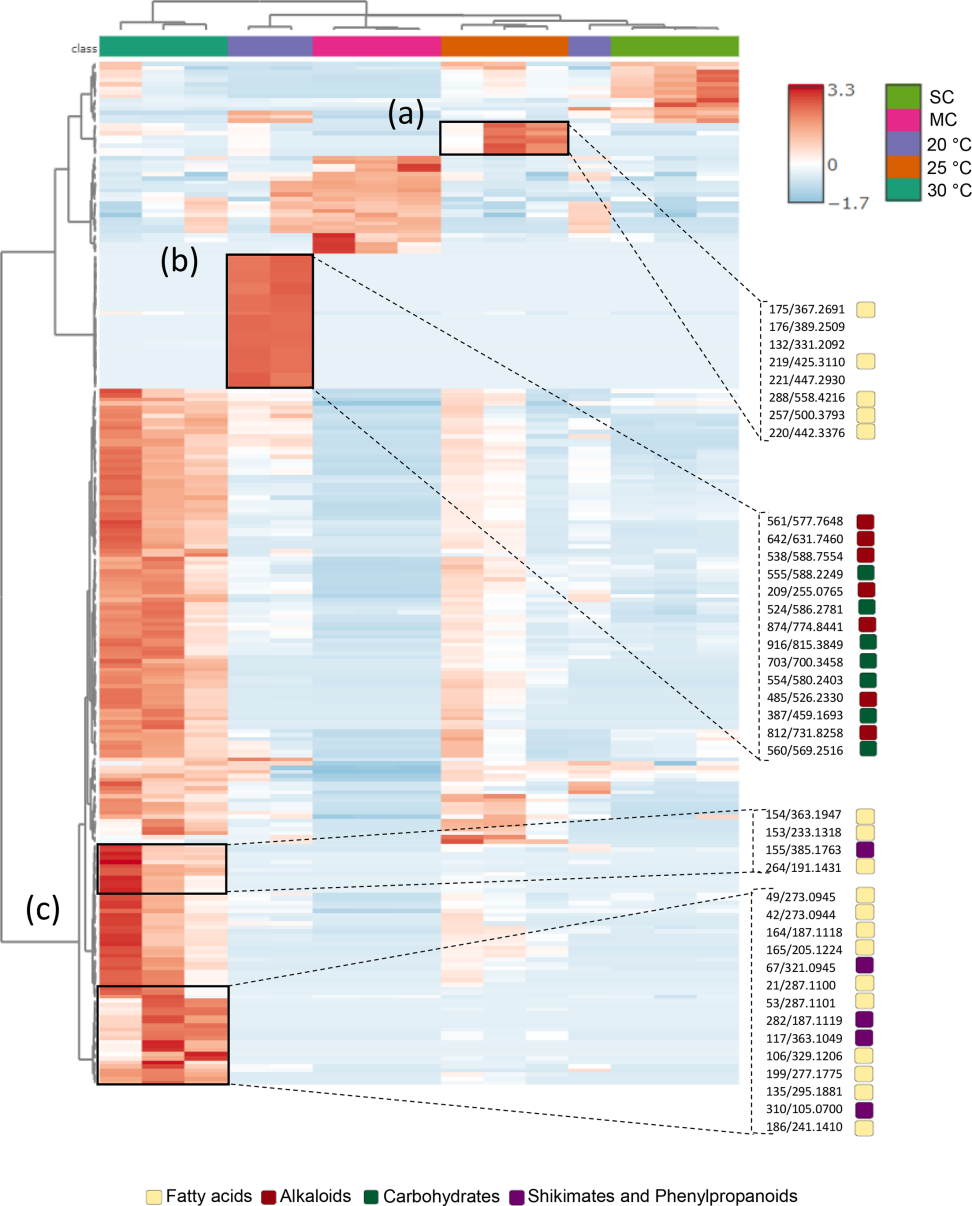
Hierarchical cluster and heat map of KRD197 metabolites when cultured at 30, 25 and 20 °C (SC, solvent control; MC, media control) ranked by ANOVA. Each row represents a metabolite feature, and each column represents a sample condition (in triplicate). The top 250 metabolite features that vary significantly (*P*<0.05) across the 3 temperatures are shown and clustered. The red colour of the tile indicates high abundance, and blue indicates low abundance. Highlighted in (**a**), (**b**) and (**c**) are features (*m/z*) of interest at each temperature with coloured boxes, indicating chemical classes.

The metabolomic profile of *Rhodococcus* KRD197 varied significantly with temperature. Hierarchical clustering of the top 250 features showed that profiles grouped primarily by temperature, with biological replicates displaying similar profiles (*P*>0.05), while significant differences were observed between temperatures and controls (*P*<0.05, Fig. 4). The heat map further revealed temperature-specific metabolite patterns: at 30 °C, features linked to fatty acid pathways were predominant; at 25 °C, only a small set of fatty acid-related features was present; and at 20 °C, an enrichment of carbohydrate pathways was observed. However, most features were shared between 30 and 25 °C, indicating a more distinct metabolic shift at 20 °C.

Volcano plot analysis confirmed these trends, highlighting significant metabolite abundance changes across temperatures (*P*<0.1, fold change >2.0). Although FBMN indicated an overall increase in the number of features at lower temperatures, volcano plots revealed that higher temperatures (30 °C) led to a greater number of significantly increased metabolites (163 features at 30 °C vs. 4 features at 20 °C, Fig. S8B). Notably, 19% of the elevated features were associated with fatty acid metabolism, and features that vary with temperature accounted for 65% of the total (163 of 250), suggesting a substantial shift in specialized metabolite production driven by temperature changes.

### Temperature-dependent production of antimicrobial compounds

Through molecular networking and dereplication using GNPS and DEREPLICATOR, several metabolites were annotated, including cyclo-(Leu-Phe) and cyclo-(Val-Phe), among others. To support these annotations, additional analysis was performed using SIRIUS [23], which validated the possible presence of compounds such as cyclo-(Leu-Phe) (C₁₅H₂₀N₂O₂, [M+H]+, median mass error: –0.300 p.p.m.) and cyclo-(l-Val-l-Leu) (C₁₁H₂₀N₂O₂, [M+H]+, median mass error: 1.290 p.p.m.). Notably, the analysis also revealed the presence of fatty acids such as lauric acid (C₁₂H₂₄O₂, [M+H]+, median mass error: 0.448 p.p.m.) and 8-methylhexadecanoic acid (C₁₇H₃₄O₂, [M+H]+, median mass error: 1.545 p.p.m.) (Fig. S9), detected exclusively in strain KRD197 extract cultured at 20 °C. Lauric acid, in particular, has previously been associated with antimicrobial activity [65]. For most other metabolites, no significant matches were found in public databases, suggesting that these strains may produce structurally diverse or novel metabolites. However, since this annotation was based solely on computational predictions and no authentic standard was used for confirmation, the identification remains tentative and further experimental validation would be necessary to confirm the presence of these metabolites.

## Discussion

The phylogenetic analysis led to the selection of five *Rhodococcus* strains, whose growth was characterized through OD measurements at 600 nm. The growth curves showed expected phases, with stationary phases reached between 48 and 132 h at 30 and 25 °C. Similar growth behaviours have been observed by *Rhodococcus* sp. NAM 81 (stationary phase at 42 h [66]) and *R. opacus* MITGM-173 (72 h [67]). *R. fascians*, in particular, exhibited growth patterns consistent with previous reports, where the stationary phase was reached on the third day at an OD_600_ of ~1.75 in glycerol medium [68]. Given that secondary metabolite production is commonly associated with the stationary phase, metabolite extraction was standardized to occur 24 h after the onset of the stationary phase. This approach is supported by previous studies that reported peak antibiotic production during this phase, for instance, *Streptomyces coelicolor* producing undecylprodigiosin and actinorhodin [69] and *Streptomyces goldiniensis* producing aurodox [70].

Metabolomic analysis revealed that *Rhodococcus* KRD197 exhibits a temperature-dependent metabolite profile, with a notable increase in metabolic diversity at lower temperatures. This shift was characterized by a significant enrichment of carbohydrate-associated features, a pattern consistent with studies on psychrotolerant fungi (*Atradidymella* sp.), which showed increased metabolite production at 4 °C [71]. In other micro-organisms, such as algae, an increased carbohydrate output has been associated with osmotic stress regulation, as observed from *Chlamydomonas reinhardtii*, where extracellular carbohydrate and polymeric substance production contribute to cold adaptation [72,73]. In this study, temperature-dependent metabolic modulation was also observed, and branched-chain fatty acids with reported antimicrobial activity were detected exclusively at 20 °C, which could indicate that cold stress could also trigger biosynthetic pathways related to antimicrobial production. Branched-chain fatty acids have been reported to disrupt bacterial membranes, inhibit protein synthesis and affect gene replication [74]. These findings suggest that cold tolerance in KRD197 is a complex process, potentially integrating osmotic regulation, membrane stability and antimicrobial metabolite production as adaptive mechanisms.

In contrast, higher temperatures (particularly at 30 °C) exhibited a significant increase in fatty acids, which – though typically considered primary metabolites – may play adaptive roles under thermal stress. This is consistent with findings from *Caenorhabditis elegans*, where fatty acid metabolism plays a role in heat resistance, osmotic balance and oxidative stress response [75]. The composition of membrane fatty acids is tightly regulated by temperature, with cold stress promoting increased unsaturation to maintain membrane fluidity, while thermophilic bacteria exhibit low unsaturated fatty acid content to enhance membrane stability [76,79].

In summary, *Rhodococcus* KRD197 appears to exhibit a dual metabolic adaptation strategy in response to temperature. At lower temperatures, the strain showed increased carbohydrate production and the possible selective biosynthesis of antimicrobial metabolite, suggesting roles in osmotic regulation and chemical defence. Conversely, at higher temperatures, a shift towards fatty acid metabolism likely contributed to membrane stability and enhanced stress tolerance. These findings provide insight into the temperature-driven metabolic plasticity of *Rhodococcus* KRD197 and highlight its biotechnological potential in diverse and variable environmental conditions.

While this study provides a comprehensive metabolomic profile of *Rhodococcus* KRD197, future research should focus on integrating genomic and metabolomic approaches to further elucidate its biosynthetic capabilities. Whole-genome sequencing and comparative genomic analyses could facilitate the identification of BGCs associated with the production of specialized metabolites, particularly those that are temperature regulated, thereby advancing our understanding of the genetic basis for environmental adaptation and natural product biosynthesis in this promising strain.

## Supplementary material

10.1099/mic.0.001598 Uncited Supplementary Material 1.

## Abbreviations

BGC: biosynthetic gene cluster
DS: dry/stamp
FBMN: Feature-Based Molecular Networking
LC-HRMS/MS: liquid chromatography-high-resolution MS
ML: maximum likelihood
MP: maximum parsimony
NA: nutrient agar
NCBI: National Center for Biotechnology Information
SC: starch casein

## References

[1] Salam MdA, Al-Amin MdY, Salam MT, Pawar JS, Akhter N (2023). Antimicrobial resistance: a growing serious threat for global public health. Healthcare.

[2] Ranjbar R, Alam M, Antimicrobial Resistance Collaborators (2022). Global burden of bacterial antimicrobial resistance in 2019: a systematic analysis. Evid Based Nurs.

[3] Hutchings MI, Truman AW, Wilkinson B (2019). Antibiotics: past, present and future. Curr Opin Microbiol.

[4] Alam K, Mazumder A, Sikdar S, Zhao Y-M, Hao J (2022). Streptomyces: the biofactory of secondary metabolites. Front Microbiol.

[5] Embley TM, Stackebrandt E (1994). The molecular phylogeny and systematics of the actinomycetes. Annu Rev Microbiol.

[6] Schneider YK (2021). Bacterial natural product drug discovery for new antibiotics: strategies for tackling the problem of antibiotic resistance by efficient bioprospecting. Antibiotics.

[7] Hochlowski JE, Swanson SJ, Ranfranz LM, Whittern DN, Buko AM (1987). Tiacumicins, a novel complex of 18-membered macrolides. II. Isolation and structure determination. J Antibiot.

[8] Medema MH, Kottmann R, Yilmaz P, Cummings M, Biggins JB (2015). Minimum information about a biosynthetic gene cluster. Nat Chem Biol.

[9] Ceniceros A, Dijkhuizen L, Petrusma M, Medema MH (2017). Genome-based exploration of the specialized metabolic capacities of the genus *Rhodococcus*. BMC Genom.

[10] Undabarrena A, Valencia R, Cumsille A, Zamora-Leiva L, Castro-Nallar E (2021). *Rhodococcus* comparative genomics reveals a phylogenomic-dependent non-ribosomal peptide synthetase distribution: insights into biosynthetic gene cluster connection to an orphan metabolite. Microb Genom.

[11] Gavriilidou A, Kautsar SA, Zaburannyi N, Krug D, Müller R (2022). Compendium of specialized metabolite biosynthetic diversity encoded in bacterial genomes. Nat Microbiol.

[12] Cappelletti M, Presentato A, Piacenza E, Firrincieli A, Turner RJ (2020). Biotechnology of *Rhodococcus* for the production of valuable compounds. Appl Microbiol Biotechnol.

[13] Iwatsuki M, Tomoda H, Uchida R, Gouda H, Hirono S (2006). Lariatins, antimycobacterial peptides produced by *Rhodococcus* sp. K01-B0171, have a lasso structure. J Am Chem Soc.

[14] Iwatsuki M, Uchida R, Takakusagi Y, Matsumoto A, Jiang C-L (2007). Lariatins, novel anti-mycobacterial peptides with a lasso structure, produced by *Rhodococcus jostii* K01-B0171. J Antibiot.

[15] Zampolli J, De Giani A, Di Canito A, Sello G, Di Gennaro P (2022). Identification of a novel biosurfactant with antimicrobial activity produced by *Rhodococcus opacus* R7. Microorganisms.

[16] Sayers EW, Beck J, Bolton EE, Brister JR, Chan J (2025). Database resources of the National Center for Biotechnology Information in 2025. Nucleic Acids Res.

[17] Blin K, Shaw S, Medema MH, Weber T (2024). The antiSMASH database version 4: additional genomes and BGCs, new sequence-based searches and more. Nucleic Acids Res.

[18] Millán-Aguiñaga N, Soldatou S, Brozio S, Munnoch JT, Howe J (2019). Awakening ancient polar *Actinobacteria*: diversity, evolution and specialized metabolite potential. Microbiology.

[19] Parra J, Soldatou S, Rooney LM, Duncan KR (2021). *Pseudonocardia abyssalis* sp. nov. and *Pseudonocardia oceani* sp. nov., two novel actinomycetes isolated from the deep Southern Ocean. Int J Syst Evol Microbiol.

[20] Silva LJ, Crevelin EJ, Souza DT, Lacerda-Júnior GV, de Oliveira VM (2020). Actinobacteria from Antarctica as a source for anticancer discovery. Sci Rep.

[21] Martinet L, Naômé A, Deflandre B, Maciejewska M, Tellatin D (2019). A single biosynthetic gene cluster is responsible for the production of bagremycin antibiotics and ferroverdin iron chelators. mBio.

[22] Nothias L-F, Petras D, Schmid R, Dührkop K, Rainer J (2020). Feature-based molecular networking in the GNPS analysis environment. Nat Methods.

[23] Dührkop K, Fleischauer M, Ludwig M, Aksenov AA, Melnik AV (2019). SIRIUS 4: a rapid tool for turning tandem mass spectra into metabolite structure information. Nat Methods.

[24] Pang Z, Lu Y, Zhou G, Hui F, Xu L (2024). MetaboAnalyst 6.0: towards a unified platform for metabolomics data processing, analysis and interpretation. Nucleic Acids Res.

[25] Riccardi C, Calvanese M, Ghini V, Alonso-Vásquez T, Perrin E (2023). Metabolic robustness to growth temperature of a cold- adapted marine bacterium. mSystems.

[26] Jensen PR, Gontang E, Mafnas C, Mincer TJ, Fenical W (2005). Culturable marine actinomycete diversity from tropical Pacific Ocean sediments. Environ Microbiol.

[27] Supong K, Suriyachadkun C, Tanasupawat S, Suwanborirux K, Pittayakhajonwut P (2013). *Micromonospora sediminicola* sp. nov., isolated from marine sediment. Int J Syst Evol Microbiol.

[28] Shirling EB, Gottlieb D (1966). Methods for characterization of *Streptomyces* species. Int J Syst Bacteriol.

[29] Heatley NG (1944). A method for the assay of penicillin. Biochem J.

[30] Forbes BA, Sahm DF, Weissfeld AS, Trevino EA, Baron EJ, Peterson LR, Finegold SM (1990). Bailey Scotts Diagnostic Microbiology.

[31] Jiménez-Esquilín AE, Roane TM (2005). Antifungal activities of actinomycete strains associated with high-altitude sagebrush rhizosphere. J Ind Microbiol Biotechnol.

[32] Kieser T, Bibb MJ, Chater KF, Butter M, Hopwood D. (2000). Practical Streptomyces Genetics: A Laboratory Manual.

[33] Feeney MA, Newitt JT, Addington E, Algora-Gallardo L, Allan C (2022). ActinoBase: tools and protocols for researchers working on *Streptomyces* and other filamentous actinobacteria. Microb Genom.

[34] Gontang EA, Fenical W, Jensen PR (2007). Phylogenetic diversity of gram-positive bacteria cultured from marine sediments. Appl Environ Microbiol.

[35] Becerril-Espinosa A, Freel KC, Jensen PR, Soria-Mercado IE (2013). Marine Actinobacteria from the Gulf of California: diversity, abundance and secondary metabolite biosynthetic potential. Antonie Van Leeuwenhoek.

[36] Hall TA (1999). BioEdit: a user-friendly biological sequence alignment editor and analysis program for Windows 95/98/NT. Nucleic Acids Symp Ser.

[37] (1988). National Center for Biotechnology Information (NCBI). https://www.ncbi.nlm.nih.gov/.

[38] Meier-Kolthoff JP, Carbasse JS, Peinado-Olarte RL, Göker M (2022). TYGS and LPSN: a database tandem for fast and reliable genome-based classification and nomenclature of prokaryotes. Nucleic Acids Res.

[39] Meier-Kolthoff JP, Hahnke RL, Petersen J, Scheuner C, Michael V (2014). Complete genome sequence of DSM 30083(T), the type strain (U5/41(T)) of *Escherichia coli*, and a proposal for delineating subspecies in microbial taxonomy. Stand Genomic Sci.

[40] Edgar RC (2004). MUSCLE: multiple sequence alignment with high accuracy and high throughput. Nucleic Acids Res.

[41] Stamatakis A (2014). RAxML version 8: a tool for phylogenetic analysis and post-analysis of large phylogenies. Bioinformatics.

[42] Goloboff PA, Farris JS, Nixon KC (2008). TNT, a free program for phylogenetic analysis. Cladistics.

[43] Pattengale ND, Alipour M, Bininda-Emonds ORP, Moret BME, Stamatakis A (2010). How many bootstrap replicates are necessary?. J Comput Biol.

[44] Swofford D PAUP*. Phylogenetic analysis using parsimony (*and other methods). Version 4.0b102002.

[45] Rambaut A, Drummond AJ (2009). FigTree version 1.3. 1. http://tree.bio.ed.ac.uk/software/figtree/.

[46] Kaufmann KW (1981). Fitting and using growth curves. Oecologia.

[47] Motulsky H, Christopoulos A (2004). Fitting Models to Biological Data Using Linear and Nonlinear Regression: A Practical Guide to CurveFitting.

[48] Soldatou S, Eldjárn GH, Ramsay A, van der Hooft JJJ, Hughes AH (2021). Comparative metabologenomics analysis of polar actinomycetes. Marine Drugs.

[49] Chambers MC, Maclean B, Burke R, Amodei D, Ruderman DL (2012). A cross-platform toolkit for mass spectrometry and proteomics. Nat Biotechnol.

[50] Schmid R, Heuckeroth S, Korf A, Smirnov A, Myers O (2023). Integrative analysis of multimodal mass spectrometry data in MZmine 3. Nat Biotechnol.

[51] Myers OD, Sumner SJ, Li S, Barnes S, Du X (2017). One step forward for reducing false positive and false negative compound identifications from mass spectrometry metabolomics data: new algorithms for constructing extracted ion chromatograms and detecting chromatographic peaks. Anal Chem.

[52] Willforss J, Chawade A, Levander F (2019). NormalyzerDE: online tool for improved normalization of omics expression data and high-sensitivity differential expression analysis. J Proteome Res.

[53] Ewald JD, Zhou G, Lu Y, Kolic J, Ellis C (2024). Web-based multi-omics integration using the Analyst software suite. Nat Protoc.

[54] Wang M, Carver JJ, Phelan VV, Sanchez LM, Garg N (2016). Sharing and community curation of mass spectrometry data with Global Natural Products Social Molecular Networking. Nat Biotechnol.

[55] Horai H, Arita M, Kanaya S, Nihei Y, Ikeda T (2010). MassBank: a public repository for sharing mass spectral data for life sciences. J Mass Spectrom.

[56] Mohimani H, Gurevich A, Shlemov A, Mikheenko A, Korobeynikov A (2018). Dereplication of microbial metabolites through database search of mass spectra. Nat Commun.

[57] Shannon P, Markiel A, Ozier O, Baliga NS, Wang JT (2003). Cytoscape: a software environment for integrated models of biomolecular interaction networks. Genome Res.

[58] Hoffmann MA, Nothias L-F, Ludwig M, Fleischauer M, Gentry EC (2022). High-confidence structural annotation of metabolites absent from spectral libraries. Nat Biotechnol.

[59] Dührkop K, Nothias L-F, Fleischauer M, Reher R, Ludwig M (2021). Systematic classification of unknown metabolites using high-resolution fragmentation mass spectra. Nat Biotechnol.

[60] Kim HW, Wang M, Leber CA, Nothias L-F, Reher R (2021). NPClassifier: a deep neural network-based structural classification tool for natural products. J Nat Prod.

[61] Yarza P, Spröer C, Swiderski J, Mrotzek N, Spring S (2013). Sequencing Orphan Species initiative (SOS): filling the gaps in the 16S rRNA gene sequence database for all species with validly published names. Syst Appl Microbiol.

[62] Ludwig W, Viver T, Westram R, Francisco Gago J, Bustos-Caparros E (2021). Release LTP_12_2020, featuring a new ARB alignment and improved 16S rRNA tree for prokaryotic type strains. Syst Appl Microbiol.

[63] Santoyo-Garcia JH, Walls LE, Nowrouzi B, Galindo-Rodriguez GR, Ochoa-Villarreal M (2022). In situ solid-liquid extraction enhances recovery of taxadiene from engineered *Saccharomyces cerevisiae* cell factories. Sep Purif Technol.

[64] Bogdanov A, Salib MN, Chase AB, Hammerlindl H, Muskat MN (2024). Small molecule in situ resin capture provides a compound first approach to natural product discovery. Nat Commun.

[65] Matsue M, Mori Y, Nagase S, Sugiyama Y, Hirano R (2019). Measuring the antimicrobial activity of lauric acid against various bacteria in human gut microbiota using a new method. Cell Transplant.

[66] Mohammad Nawawi N, Ahmad A, Nallapan Maniyam M, Ibrahim A (2016). Biotransformation of phenol by the resting cells of *Rhodococcus* sp. NAM 81. Indian J Fundam Appl Life Sci.

[67] Kurosawa K, Radek A, Plassmeier JK, Sinskey AJ (2015). Improved glycerol utilization by a triacylglycerol-producing *Rhodococcus opacus* strain for renewable fuels. Biotechnol Biofuels.

[68] Vereecke D, Cornelis K, Temmerman W, Jaziri M, Van Montagu M (2002). Chromosomal locus that affects pathogenicity of *Rhodococcus fascians*. J Bacteriol.

[69] Manteca A, Alvarez R, Salazar N, Yagüe P, Sanchez J (2008). Mycelium differentiation and antibiotic production in submerged cultures of *Streptomyces coelicolor*. Appl Environ Microbiol.

[70] E McHugh R, Giard J, Braes RE, McKean I, Roe AJ (2023). Optimisation of aurodox production by *Streptomyces goldiniensis*. Access Microbiol.

[71] Ulaganathan Y, Weber J-F, Convey P, Rizman-Idid M, Alias SA (2017). Antimicrobial properties and the influence of temperature on secondary metabolite production in cold environment soil fungi. Polar Sci.

[72] Aslam SN, Cresswell-Maynard T, Thomas DN, Underwood GJC (2012). Production and characterization of the Intra- and Extracellular Carbohydrates and Polymeric Substances (EPS) of three sea-ice diatom species, and evidence for a cryoprotective role for EPS. J Phycol.

[73] Liu W, Cong B, Lin J, Liu S, Deng A (2023). Taxonomic identification and temperature stress tolerance mechanisms of *Aequorivita marisscotiae* sp. nov. *Commun Biol*.

[74] Casillas-Vargas G, Ocasio-Malavé C, Medina S, Morales-Guzmán C, Del Valle RG (2021). Antibacterial fatty acids: an update of possible mechanisms of action and implications in the development of the next-generation of antibacterial agents. Prog Lipid Res.

[75] Horikawa M, Sakamoto K (2009). Fatty-acid metabolism is involved in stress-resistance mechanisms of Caenorhabditis elegans. Biochem Biophys Res Commun.

[76] Zhang YM, Rock CO (2008). Membrane lipid homeostasis in bacteria. Nat Rev Microbiol.

[77] Marr AG, Ingraham JL (1962). Effect of temperature on the composition of fatty acids in *Escherichia coli*. J Bacteriol.

[78] Aguilar PS, Cronan JE, de Mendoza D (1998). A *Bacillus subtilis* gene induced by cold shock encodes a membrane phospholipid desaturase. J Bacteriol.

[79] Nordström KM, Laakso SV (1992). Effect of growth temperature on fatty acid composition of ten thermus strains. Appl Environ Microbiol.

