# Supplementary material for: Assessing the effect of temperature on Rhodococcus metabolite production
Source: Microbiology (Reading). 2025 Aug 26;171(8):001598. doi: 10.1099/mic.0.001598 (PMC12380520; doi:10.1099/mic.0.001598)
Supplement: Uncited Supplementary Material 1. [file mic-171-01598-s001.pdf]

SUPPLEMENTARY MATERIAL

Table S1. Seven *Rhodococcus* strains selected for analysis, showing: isolation location, GPS coordinates and isolation media used.

STRAIN ID	ISOLATION LOCATION	GPS Coordinates	ISOLATION MEDIA	Reference
KRD162 MH725318.1	Antarctic	62 57.68S, 27 53.51W	A1	Mincer, *et al*. 2002
KRD175 MH725305.1	Arctic	79 00.01N, 06 56.84E	A1	Mincer, *et al*. 2002
KRD196 MH725284.1	Antarctic	62 11.420S, 49 29.45W	A1	Mincer, *et al.* 2002
KRD197 MH725283.1	Antarctic	58 16.15S, 24 54.26W	A1	Mincer, *et al*. 2002
KRD207	Antarctic	62 57.68S, 27 53.51W	SC	Mohseni, *et al*. 2013
KRD226	Scotland	-56° 27’ 13” N, 5° 23’ 39” W	SC	Mohseni, *et al*. 2013
KRD231	Scotland	- 56° 29’ 24” N, 5° 26’ 37” W	SC	Mohseni, *et al*. 2013

Table S2. *Rhodococcus* strains selected for phylogenetic analysis with NCBI accession numbers, percent identity (%ID), query cover (%QC) and sequence lenght to each study strain (KRD).

Strain	NCBI reference strains with accession number	Percent identity	Query cover	Sequence length
KRD162				
	Rhodococcus sovatensis NR_156055.1	99.37	99	1414
	Rhodococcus yunnanensis NR_043009.1	99.06	98	1466
	Rhodococcus cercidiphylli NR_116275.1	98.96	98	1480
	Rhodococcus jostii NR_118421.1	97.49	99	1420
	Rhodococcus spelaei NR_180470.1	96.04	99	1429
KRD175				
	Rhodococcus fascians NR_037021.1	99.89	99	1424
	Rhodococcus yunnanensis NR_043009.1	99.56	100	1466
	Rhodococcus cercidiphylli NR_116275.1	99.45	100	1480
	Nocardia globerula NR_104795.1	97.01	100	1507
	Rhodococcus pedocola NR_149270.1	96.46	99	1472
KRD196				
	Rhodococcus kyotonensis NR_041512.1	99.02	99	1420
	Rhodococcus yunnanensis NR_043009.1	98.91	99	1466
	Rhodococcus sovatensis NR_156055.1	98.37	99	1414
	Rhodococcus qingshengii NR_115708.1	97.29	99	1489
	Rhodococcus spelaei NR_180470.1	96.19	99	1429
KRD197				
	Rhodococcus yunnanensis NR_043009.1	98.91	100	1466
	Rhodococcus cercidiphylli NR_116275.1	98.80	100	1480
	Rhodococcus kyotonensis NR_041512.1	99.22	98	1420
	Rhodococcus jostii NR_118421.1	97.06	100	1420
	Rhodococcus olei NR_179708.1	96.07	100	1455
KRD207				
	Rhodococcus cerastii NR_117103.1	99.56	97	1337
	Rhodococcus sovatensis NR_156055.1	99.24	99	1414
	Rhodococcus cercidiphylli NR_116275.1	99.03	100	1480
	Rhodococcus koreensis NR_024973.1	97.28	100	1473
	Rhodococcus nanhaiensis NR_109481.1	96.09	99	1400
KRD226				
	Nocardia coeliaca NR_104776.1	99.89	100	1507
	Rhodococcus qingshengii NR_115708.1	99.89	100	1489
	Rhodococcus erythropolis NR_037024.1	99.89	100	1476
	Rhodococcus jostii NR_118421.1	98.12	100	1420
	Rhodococcus oryzae NR_170410.1	97.00	100	1474
KRD231				
	Nocardia coeliaca NR_104776.1	98.47	100	1507
	Rhodococcus jostii NR_118421.1	98.40	100	1420
	Rhodococcus spelaei NR_180470.1	97.02	99	1429
	Rhodococcus corynebacterioides NR_041873.1	96.01	100	1494
	Rhodococcus kyotonensis NR_041512.1	95.56	99	1420

(A)

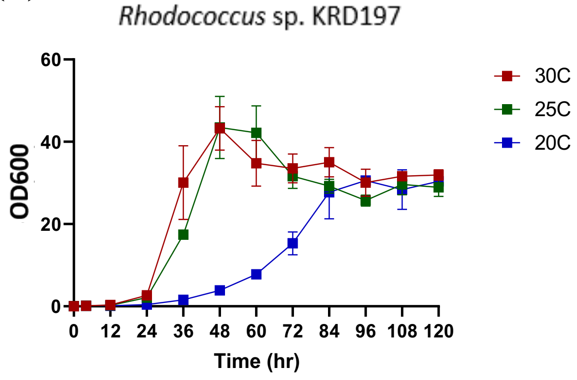

(B)

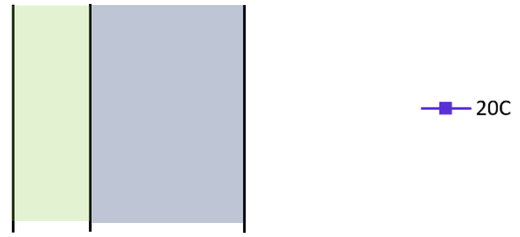

Figure S1. Cell density over time for *Rhodococcus* KRD197 grown at 20 °C, 25 °C, and 30 °C in ISP2 medium. Data points represent the mean of three biological replicates, and error bars indicate the standard deviation. (A) Growth curve: Time (hours) is plotted on the x-axis, and optical density at 600 nm (OD_{600}) on the y-axis. (B) Logarithmic growth curve: Time (hours) is on the x-axis, and the logarithmic scale of OD_{600} is on the y-axis. Green and blue shading highlight the exponential growth phase, occurring between 12–36 h at 30 °C and 25 °C, and 12–84 h at 20 °C, respectively.

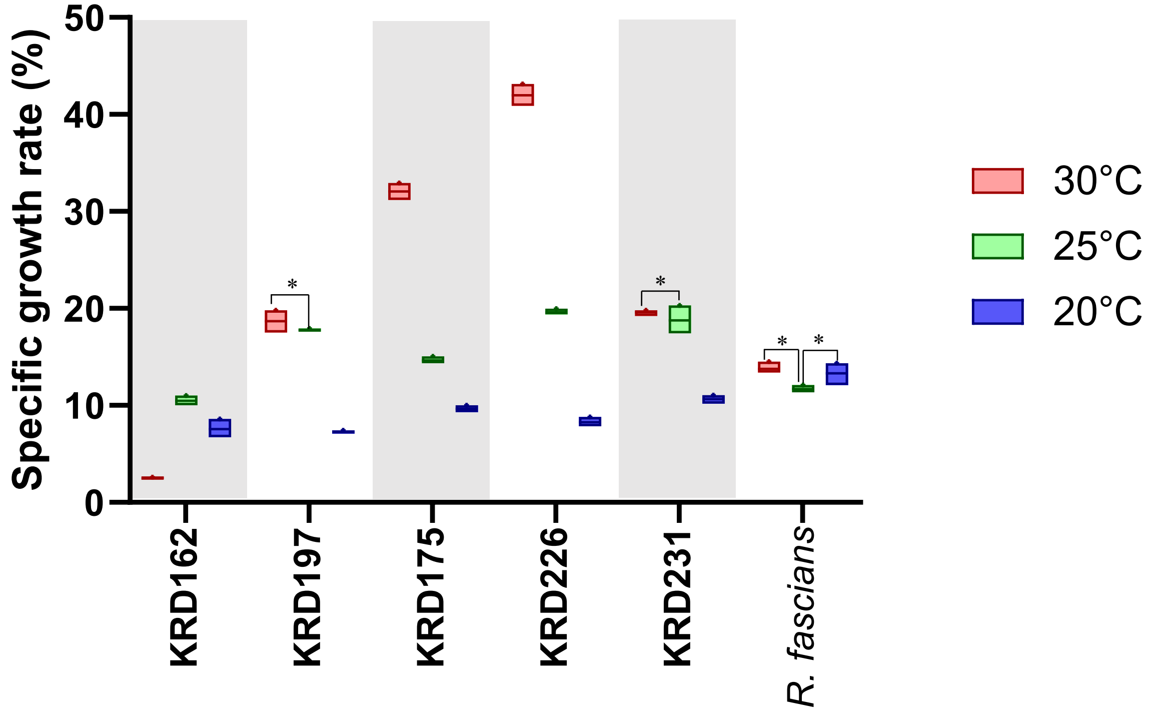

Figure S2. Box plots showing the specific growth rates of the six *Rhodococcus* strains (KRD162, 197, 175, 226, 231 and *R. fascians*) grown at three different temperatures: 30 °C (Red), 25 °C (green) and 20 °C (blue). Boxplots show the range from 5 to 95% with the mean values as thick lines. P values are based on a between-temperatures t test. The asterisk and the lines mark the temperatures that do **NOT** show significant P values (P>0.05).

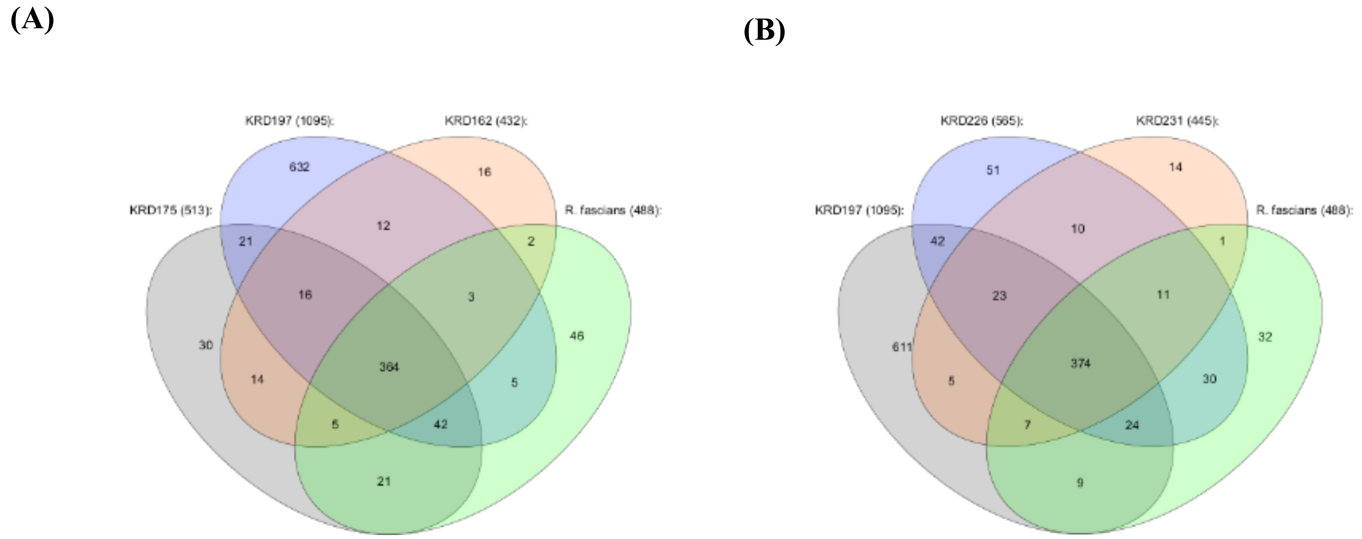

Figure S3. Euler diagram depicting the distribution of features detected across all *Rhodococcus* metabolites extracts. Specific and shared features among (A) Arctic/Antarctic *Rhodococcus* strains and (B) KRD197 compared with Scottish *Rhodococcus* strains. Specific features potentially represent distinctive or particular features from a strain, while shared features potentially mean features shared across strains.

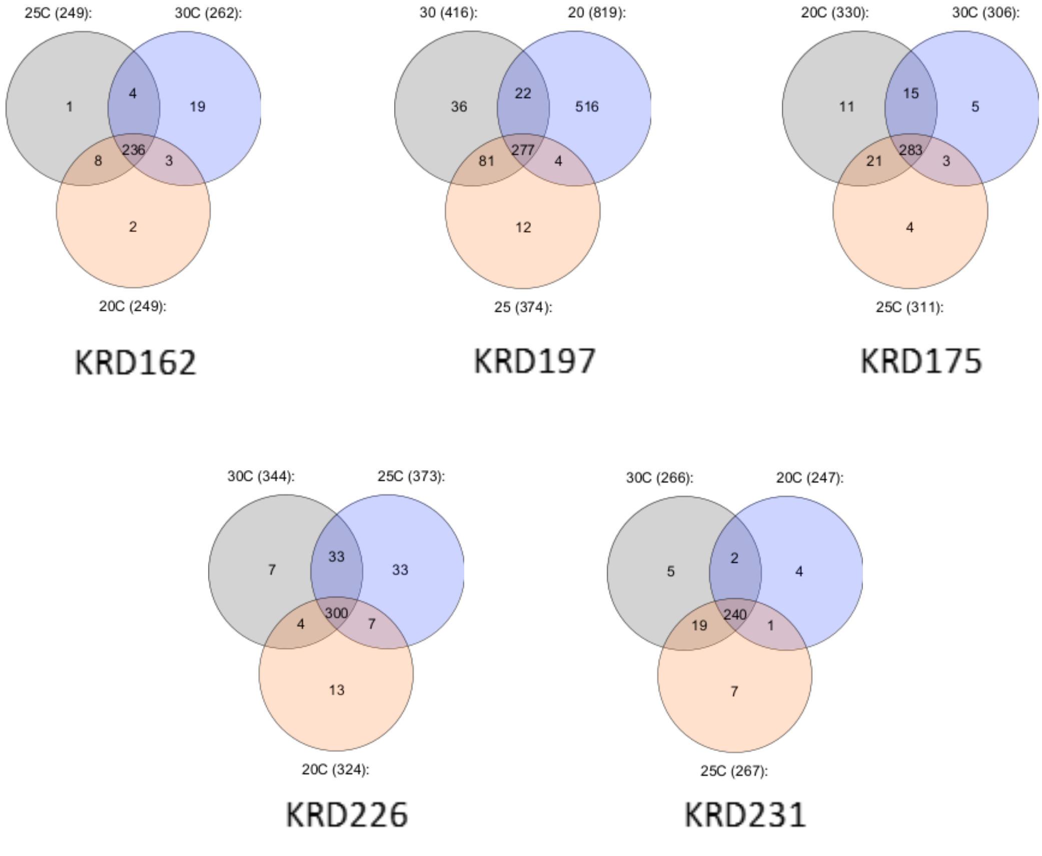

Figure S4. **Venn diagrams showing the distribution of compounds across *Rhodococcus* strains (KRD162, KRD197, KRD175, KRD226, KRD231) at three different temperatures (20 °C, 25 °C, and 30 °C).** Strain-specific and shared compounds are represented, with colours indicating temperature: grey (30 °C), pink (25 °C), and purple (20 °C).

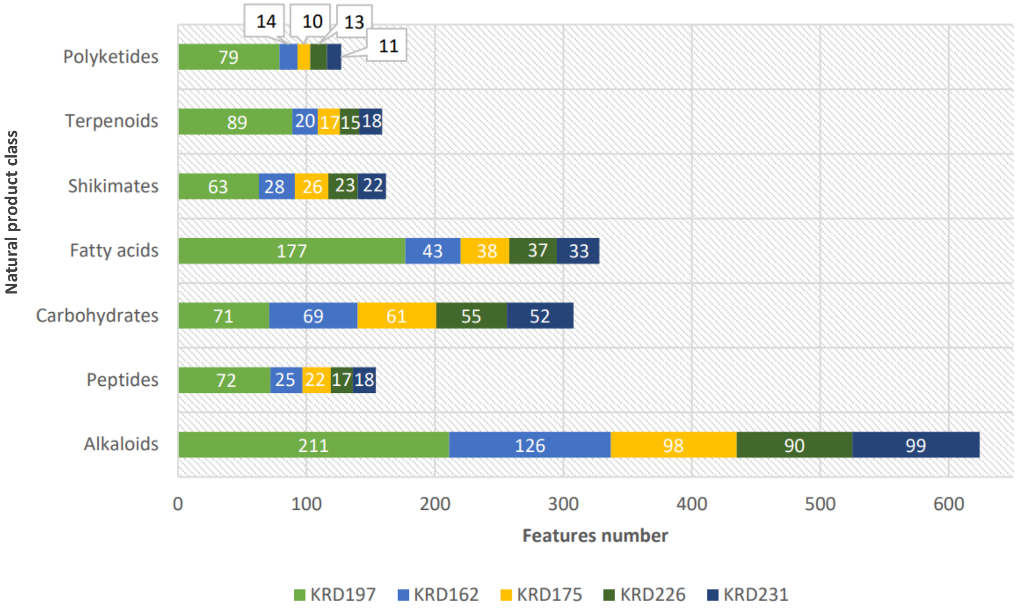

Figure S5. Number of compounds per biosynthetic pathways for all *Rhodococcus* strains analysed at different temperatures (30 °C, 25 °C, and 20 °C). The biosynthetic pathways identified include alkaloids, amino acids and peptides, carbohydrates, fatty acids, polyketides, terpenoids, and shikimates/phenylpropanoids.

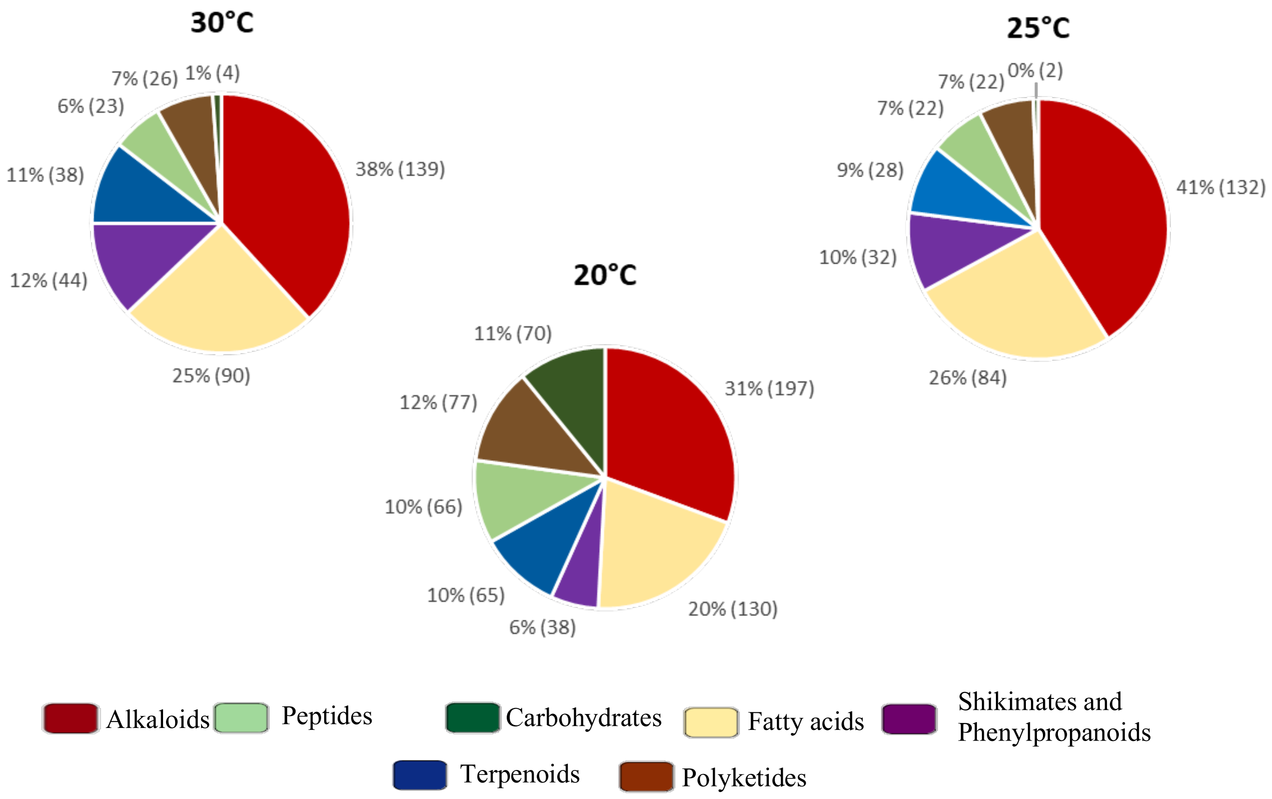

Figure S6. Pie chart illustrating the distribution of chemical classes produced by the *Rhodococcus* strain KRD197 across three incubation temperatures (20 °C, 25 °C, and 30 °C). Metabolites were categorized based on their biosynthetic pathways into alkaloids, amino acids and peptides, carbohydrates, fatty acids, polyketides, terpenoids, and shikimates/phenylpropanoids.

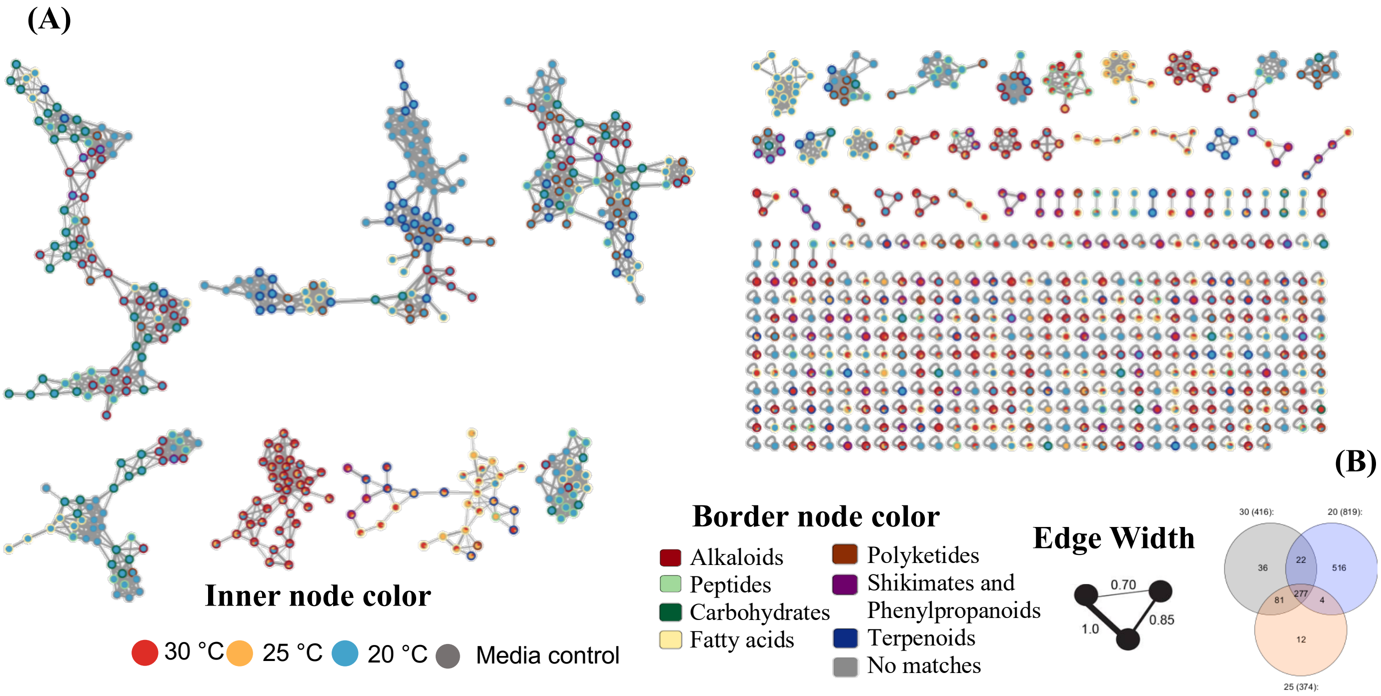

Figure S7. (A) Feature based molecular networking of *Rhodococcus* KRD197 at 20 °C, 25 °C and 30 °C. Inner pie chart node colour represents the presence of each feature across the three temperatures; grey represents features found in solvent/media controls. Border node colour represents the chemical class annotation by SIRIUS/CONAPUS, features without matches are coloured grey. (B) Venn diagram of number strain specific and shared metabolite by the KRD197 *Rhodococcus* strain across three temperatures.

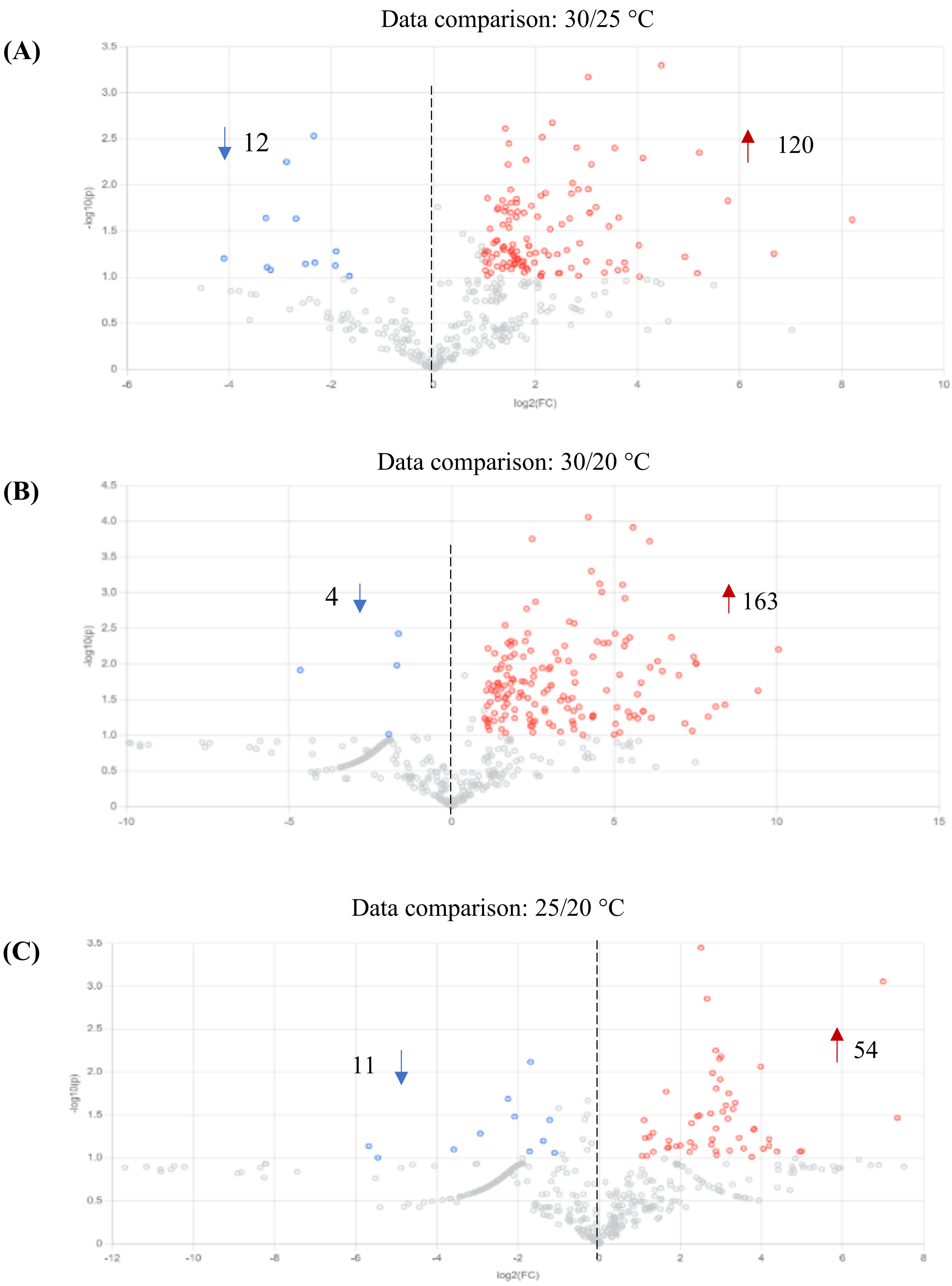

Figure S8. Volcano-plot of quantified peaks in KRD197 cultured at 20 °C, 25 °C and 30 °C. The x-axis is the mean ratio fold-change (plotted on a log 2 scale) of the relative abundance of each metabolite between the two samples selected. The y-axis represents the statistical significance p-value (threshold: 0.1) of the ratio fold-change (threshold: 2.0) for each metabolite. Red dots/arrow indicate peaks up regulated by the higher temperature compared, blue represent down regulated by higher temperature compared and numbers represents the number of features with significant change. Grey dots represent metabolites detected but that are not significantly different. (A) Comparison between 30 °C and 25 °C, (B) Comparison between 30 °C and 20 °C, and (C) Comparison between 25 °C and 20 °C.

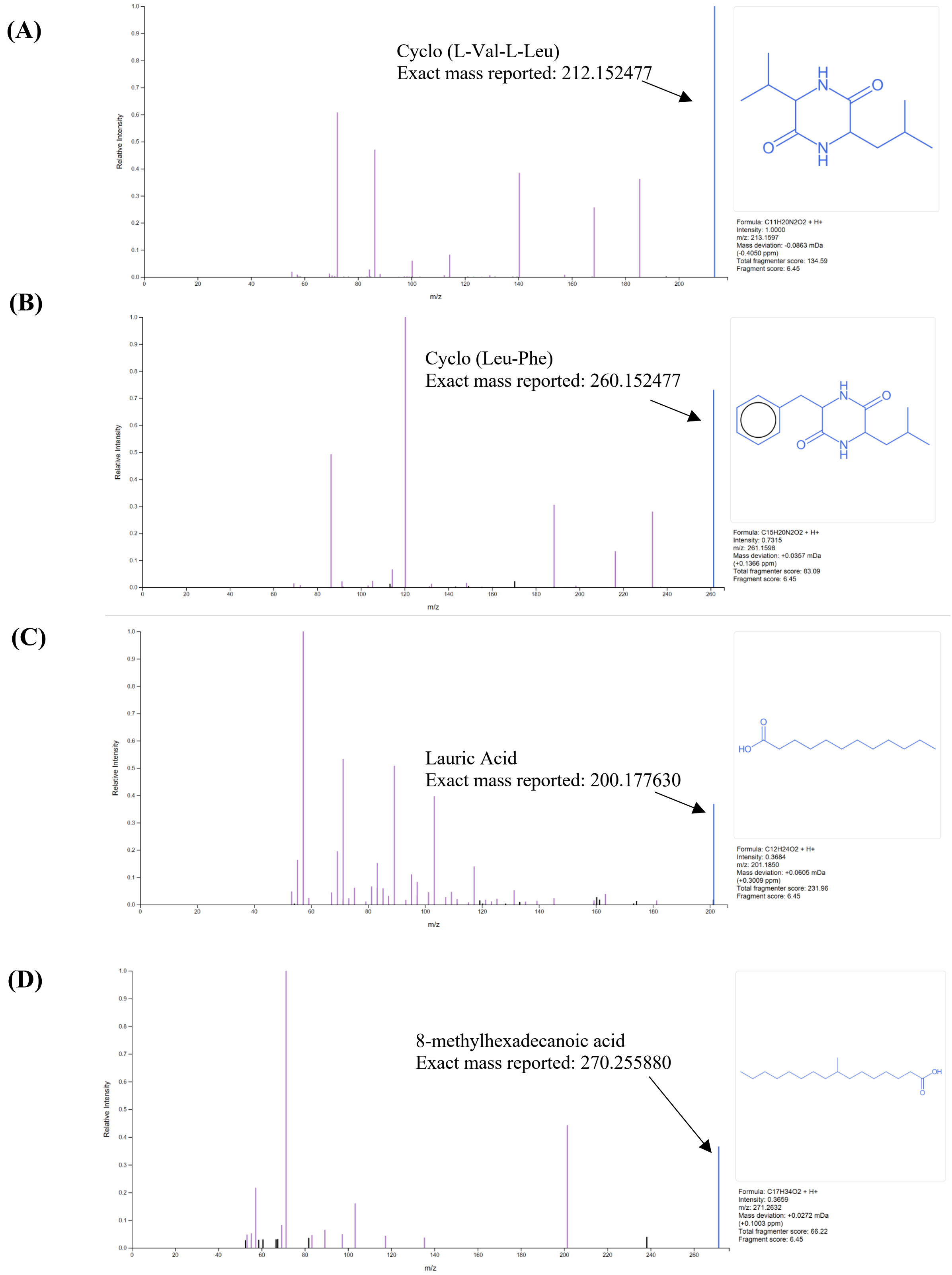

Figure S9. SIRIUS-annotated MS2 spectra acquired by UHPLC-Orbitrap MS/MS in positive mode: (a) Cyclo-(l-Val-l-Leu) (*m/z* $[M + H]^+$ 213.597), (b) Cyclo-(Leu-Phe) (*m/z* $[M + H]^+$ 261.1598), (c) Lauric Acid (*m/z* $[M + H]^+$ 201.1850) and (d) 8-methylhexadecanoic acid (*m/z* $[M + H]^+$ 271.2632). The exact mass reported of the compounds corresponds to the experimental data obtained in this work. The compound structure and the metadata below each structure (molecular formula, intensity, m/z, mass deviation in mDa, total fragmenter score, and fragment score) were predicted and annotated by SIRIUS (v5.8.6).

Note: The median mass error reported in the main text is expressed in ppm and represents the median

difference between experimental and theoretical fragment masses. The mass deviation reported by SIRIUS in this figure refers to the spread (dispersion) of these errors and is expressed in mDa.
